# Heart Rate and Prognosis of Heart Failure with Reduced Ejection Fraction in Women and Men in Sinus Rhythm

**DOI:** 10.3390/jcm14061995

**Published:** 2025-03-15

**Authors:** Antonio de Padua Mansur, Maria Eduarda Bergamo, Geovana Braga do Nascimento, Giovanna Silva Machado, Carlos Henrique Del Carlo, Solange Desirée Avakian, Antonio Carlos Pereira-Barretto, Edimar Alcides Bocchi

**Affiliations:** 1 Serviço de Prevencao, Cardiopatia na Mulher e Reabilitação Cardiovascular, Instituto do Coracao (InCor), Hospital das Clinicas HCFMUSP, Faculdade de Medicina, Universidade de Sao Paulo, Sao Paulo 05403-900, SP, Brazil; mebergamo@usp.br (M.E.B.); pereira.barreto@incor.usp.br (A.C.P.-B.); 2 Faculdade de Medicina Santa Marcelina, Sao Paulo 05006-020, SP, Brazil; geovana_braga@usp.br (G.B.d.N.); 11022120808@aluno.santamarcelina.edu.br (G.S.M.); 3 Hospital Dia, Instituto do Coracao (InCor), Hospital das Clinicas HCFMUSP, Faculdade de Medicina, Universidade de Sao Paulo, Sao Paulo 05403-900, SP, Brazil; del_carlo@uol.com.br; 4 Unidade Clinica de Valvopatias, Instituto do Coracao (InCor), Hospital das Clinicas HCFMUSP, Faculdade de Medicina, Universidade de Sao Paulo, Sao Paulo 05403-900, SP, Brazil; solange.avakian@incor.usp.br; 5 Unidade Clinica de Insuficiencia Cardiaca, Instituto do Coracao (InCor), Hospital das Clinicas HCFMUSP, Faculdade de Medicina, Universidade de Sao Paulo, Sao Paulo 05403-900, SP, Brazil; edimar.bocchi@fm.usp.br

**Keywords:** heart failure, heart rate, sinus rhythm, prognosis, women, men

## Abstract

**Background**: A resting heart rate (RHR) is a guideline-recommended therapeutic target for all patients with heart failure with reduced ejection fraction (HFrEF), with reductions to 60 bpm linked to improved outcomes. Conversely, elevated RHR is associated with increased mortality in HFrEF. However, sex-specific differences in mortality, particularly for women in sinus rhythm, remain unclear. We evaluated mortality rates at RHR thresholds of ≤60 bpm and ≤70 bpm in women and men with HFrEF. **Methods**: From February 2017 to January 2022, we assessed 2984 patients (61 ± 13.8 years, 64.4% men) with HFrEF in sinus rhythm. Clinical and echocardiographic data were analyzed to examine RHR’s influence on mortality. **Results**: Over a mean follow-up of 3.7 ± 1.6 years, left ventricular ejection fraction improved in men (29.5 ± 6.7% to 36.7 ± 12.9%; *p* < 0.001) and women (29.9 ± 6.4% to 38.0 ± 13.4%; *p* < 0.001). Men had higher mortality (43.7% vs. 36.7%; *p* < 0.001), with cumulative death incidence greater at an RHR > 60 bpm (*p* < 0.001) and >70 bpm (*p* = 0.011). Cox regression identified an RHR as an independent predictor of mortality for men (HR = 1.008; *p* = 0.008) but not women. **Conclusions**: An elevated RHR increases mortality risk in men, suggesting a target near 60 bpm and closer to 70 bpm in women, supporting individualized RHR management.

## 1. Introduction

An increased resting heart rate (RHR) has long been associated with a higher incidence of heart failure (HF) in both healthy women and men. The European Prospective Investigation into Cancer and Nutrition (EPIC) study demonstrated a graded association between the RHR and the risk of developing HF in apparently healthy individuals, indicating that an elevated RHR is a significant risk factor for HF [1]. This association remained strong even after multivariable adjustments for various covariates, suggesting that an RHR is crucial in predicting HF in men. However, the same study did not observe a similar relationship in women, which raises the question of whether the prognostic value of the RHR may differ by gender. Similarly, the Rotterdam Study also found an association between the RHR and HF in men but not women, particularly after excluding patients taking heart rate-modifying medications [2].

In contrast, the Framingham Heart Study (FHS) established a graded relationship between RHR and the risk of developing HF in both healthy women and men. The study further reinforced the idea that the baseline RHR serves as a critical predictor of both HF and cardiovascular mortality and all-cause mortality, emphasizing its importance as a prognostic tool [3]. A more recent study in a Chinese population identified a J-shaped association between the RHR and HF risk, indicating that very low and very high RHR values are linked to an increased risk of HF [4]. This finding underscores the complexity of the relationship between the RHR and HF across different populations. It suggests that an RHR may have differing implications for HF risk in diverse racial and ethnic groups.

An insidious onset and progressive deterioration of cardiac function characterize heart failure with reduced ejection fraction (HFrEF). In the early stages, patients with HFrEF are often asymptomatic, which makes early diagnosis and intervention difficult. Over time, as cardiac function declines, patients develop symptoms such as fatigue, shortness of breath, and exercise intolerance. This progressive decline in cardiac function is often accompanied by a compensatory increase in heart rate, driven by the activation of neurohormonal systems, primarily the sympathetic nervous system and the renin–angiotensin–aldosterone system [5]. These systems are activated in an attempt to maintain cardiac output and meet the body’s increasing demands. However, as the heart’s ability to pump blood effectively deteriorates, the compensatory mechanisms become overwhelmed, leading to worsening symptoms and, eventually, to clinical heart failure.

The current standard of care for patients with HFrEF revolves around inhibiting neurohormonal systems to prevent further deterioration of cardiac function [6]. Beta-blockers, which work by blocking the effects of the sympathetic nervous system, have become a cornerstone of HFrEF therapy. Clinical trials have demonstrated that beta-blockers improve survival rates and reduce hospitalizations in patients with HFrEF [7]. However, these trials have primarily included male patients, and the data on women have been limited. This gender imbalance in clinical trials has raised concerns that the results may not be adequately powered to detect the specific benefits or risks for female patients. As a result, the safety and efficacy of specific treatments, such as beta-blockers, may not be fully understood in women, potentially leading to suboptimal care for female patients with HFrEF [8].

An elevated RHR in HFrEF patients is a well-established predictor of higher mortality and poor clinical outcomes, making it a critical focus in HF management [9]. Reducing RHR to approximately 60 beats per minute (bpm) has become a common therapeutic goal in managing HFrEF as it has been associated with improved survival and reduced hospitalization rates. However, the target RHR may not be the same for women as it is for men, primarily due to the under-representation of women in randomized controlled trials. The physiological response to treatment, including heart rate reduction, may differ between men and women, and women may not need as significant a reduction in RHR to achieve optimal outcomes.

Gender disparities in the impact of the RHR on HF outcomes may influence the management of HFrEF. While the relationship between an elevated RHR and increased mortality in men with HFrEF is well established, the effects of the RHR on mortality and other prognostic factors in women remain less clear. While men typically have a target RHR of 60 bpm for managing HFrEF, women may not require such a drastic reduction in the RHR. Moreover, women with HFrEF in sinus rhythm may experience different outcomes and have different predictors of mortality associated with an RHR compared to their male counterparts. The existing literature has not fully explored these potential gender-specific differences in RHR’s effects on HFrEF outcomes, particularly in women.

This study investigated gender-specific differences in the effects of RHR targets on HF outcomes, including mortality predictors, in patients with HFrEF. By exploring how an RHR affects women differently from men, the goal is to develop more personalized and effective treatment strategies for female patients with HFrEF. The findings from this research could help refine treatment protocols and, ultimately, lead to improved care and clinical outcomes for women, ensuring that female patients receive the most appropriate and effective therapies for managing their condition. Understanding the nuanced role of an RHR in female HFrEF patients is crucial for advancing gender-tailored treatment approaches to heart failure care.

## 2. Materials and Methods

### 2.1. Study Design and Population

Between February 2017 and January 2022, we conducted a retrospective study to evaluate the impact of the RHR on all-cause mortality in patients with HFrEF in sinus rhythm. The diagnosis of sinus rhythm was confirmed by the presence of a P wave before each QRS complex, with P waves being upright in leads I and II and negative in lead aVR. The treating physicians manually performed the ECG analysis. This assessment was conducted using the available diagnostic devices at the time of the examination. Specifically, we aimed to identify predictors of death in both women and men diagnosed with HFrEF in sinus rhythm, defined by a left ventricular ejection fraction (LVEF) of 40% or less. This study population included individuals diagnosed with heart failure based on the comprehensive Framingham criteria and confirmed through echocardiographic measurements.

We collected comprehensive baseline data encompassing a variety of clinical and echocardiographic characteristics. Key clinical data included the patient’s age, body mass index (BMI), and the presence of comorbid conditions, such as diabetes, chronic kidney disease (CKD), myocardial infarction, and stroke. Diabetes was defined by a fasting blood glucose level of 126 mg/dL or higher, glycated hemoglobin levels exceeding 6.5%, or using hypoglycemic medications. Chronic kidney disease was characterized by a serum creatinine level of 2 mg/dL or greater. We also documented the number of coronary artery bypass grafts (CABG), percutaneous coronary intervention (PCI), pacemakers, implantable cardioverter-defibrillator (ICD), cardiac resynchronization therapy (CRT), transplants, and hospitalizations. Echocardiographic data included baseline and follow-up assessments of LVEF and left ventricular diastolic diameters, both important indicators of cardiac function. Two-dimensional (2D) echocardiography assessed cardiac chamber dimensions, left ventricular (LV) wall thickness, and overall cardiac morphology. M-mode echocardiography provided precise measurements of LV size and function. Left ventricular ejection fraction (LVEF) was calculated using Simpson’s biplane method of discs. Doppler echocardiography evaluated diastolic function, valvular integrity, and intracardiac hemodynamics while using the available diagnostic devices during the examination. A blinded physician performed the echocardiogram. The intra- and interobserver variability varies depending on the echocardiographic parameter. In our service, for example, a key parameter in our study, the left ventricular diastolic diameter, had an intraobserver variability of approximately 4% and an interobserver variability of 7% [10].

To assess the relationship between the RHR and mortality, we stratified the RHR into two groups: ≤60 beats per minute (bpm) and >60 bpm, as well as ≤70 bpm and >70 bpm. We then compared demographic characteristics, clinical profiles, and echocardiographic findings within each RHR group, focusing on differences between women and men. This stratified approach allowed us to examine potential sex-specific variations in the impact of the RHR on mortality and the association between an RHR and key prognostic factors. Mortality data were sourced from medical records or verified through the Federal Revenue’s website, which provided individual registration statuses [11].

We utilized the closest available RHR value to the end of the follow-up period (January 2022) or the time of death, depending on the patient’s outcome. This methodological approach, approved by the Research Ethics Committee (CAPpesq) of the Hospital das Clínicas, Faculdade de Medicina, Universidade de São Paulo (approval number: 4.436.791), enabled us to explore whether the relationship between the RHR and all-cause mortality differed by sex among patients with HFrEF in sinus rhythm.

### 2.2. Primary Endpoint

The primary outcome of our study was to evaluate the association between the RHR and all-cause mortality in women and men with HFrEF in sinus rhythm.

### 2.3. Secondary Endpoints

The secondary goals were to identify sex-specific variations in the impact of an RHR on death rates and to analyze the primary prognostic predictors related to these variations.

We investigated RHR-related differences in demographic characteristics, clinical profiles, and echocardiographic findings between women and men across different RHR cut-off values.

### 2.4. Statistical Analysis

Statistical analysis was conducted by presenting continuous variables as mean ± standard deviations and categorical variables as frequencies and percentages. The normality of the data was assessed using the Equality of Variances test. Continuous variables were compared between groups using Student’s *t*-test and analysis of variance (ANOVA), while the chi-square test was employed for categorical variables. Statistical significance was determined using a two-sided probability value of <0.05. Multiple imputation was used to impute missing baseline and follow-up LVDD values and final LVEF. Multiple imputations used the MCMC method to deal with missing data. The imputed datasets were analyzed separately and combined to produce a single result, considering the uncertainty caused by missing data. The cumulative incidence of all-cause mortality was analyzed using the Kaplan–Meier (K–M) method with Šidák adjustment for multiple comparisons *p*-values. Cox proportional hazard models identified independent predictors of all-cause mortality. Predictors were selected based on a *p*-value < 0.25 in preliminary analyses, including age, gender, BMI, baseline LVEF, and a final RHR. The chi-square statistic from the Cox model was used to determine the strongest predictors. Stratified analyses explored potential interactions between these variables and mortality. Statistical analyses were conducted using the SAS^®^ Studio software package (SAS Institute, Cary, NC, USA).

## 3. Results

We analyzed a cohort of 2984 patients, with a mean age of 61 ± 13.8 years, comprising 1922 (64.4%) male participants. Baseline characteristics, including age, BMI, history of MI, diabetes, CKD, stroke, beta-blockers, angiotensin-converting enzyme inhibitors (ACEI) or angiotensin receptor blockers (ARB), hydrochlorothiazide, carvedilol dosage, hospitalization rates, basal LVEF, pacemaker and CRT implantation, and transplant, were comparable between women and men. Spironolactone and furosemide use were higher in women. IDC implantation, CABG, and PCI were higher in men. Final LVEF was higher (38 ± 13.4 vs. 36.7 ± 12.9%; *p* = 0.016), and basal (63.3 ± 7.9 vs. 65.4 ± 8.7 mm; *p* < 0.001) and final (61.2 ± 9.9 vs. 63.1 ± 10.6 mm; *p* < 0.001) LVDD were lower in women. Throughout the follow-up period, which averaged 3.7 ± 1.6 years, we observed an increase in LVEF and a decrease in LVDD in women and men. (Table 1)

Table 2 presents RHR categorized by RHR in ≤60> bpm and ≤70> bpm. Basal LVDD was lower in patients with RHR > 60 bpm than those with HR ≤ 60 bpm (66.2 ± 8.7 mm vs. 64.6 ± 8.5 bpm; *p* = 0.041). Among the patients studied, only 4.1% achieved an RHR of ≤60 bpm while taking a mean daily dosage of 42.4 ± 15.2 mg of carvedilol, and 42.5% achieved an RHR of ≤ 70 bpm with 40.8 ± 16.2 mg. Throughout the follow-up period, we observed a higher death incidence in patients with RHR > 70 bpm compared with RHR ≤ 70 bpm (42.8% vs. 39.1%; *p* = 0.044) and an increase in final LVEF for patients with RHR ≤60> and ≤70>.

Table 3 presents data for RHR categorized by sex and ≤60 bpm and >60 bpm. RHR was similar between women and men in each categorized group. Age was higher in women with RHR ≤ 60 bpm compared to RHR > 60 bpm (65 ± 11.9 vs. 58.4 ± 13.5 years; *p* = 0.011). Women with RHR > 60 bpm had higher final LVEF and lower basal and final LVDD. Spironolactone and furosemide use was higher in women with RHR > 60 bpm. The incidence of death was higher in men with RHR > 60 bpm. There was a significant increase in final LVEF compared to basal LVEF among male and female patients with RHR ≤ 60 bpm and >60 bpm.

Table 4 presents data for RHR categorized by sex and ≤70 bpm and >70 bpm. RHR was similar between women and men in each categorized group. Women with RHR ≤ 70 bpm had higher final LVEF. Basal and final LVDD were lower in women with RHR ≤ 70 bpm and >70 bpm. Spironolactone use was higher in women with RHR > 70 bpm. CABG, PCI, and ICD implantation were higher in men, and pacemaker implantation was higher in women with RHR ≤ 70 bpm. Spironolactone use and CRT implantation were higher in women. The incidence of death was marginally higher in men with RHR ≤ 70 bpm (*p* = 0.095) and significantly higher in men with RHR > 70 bpm (*p* = 0.001).

**Figure 1:**
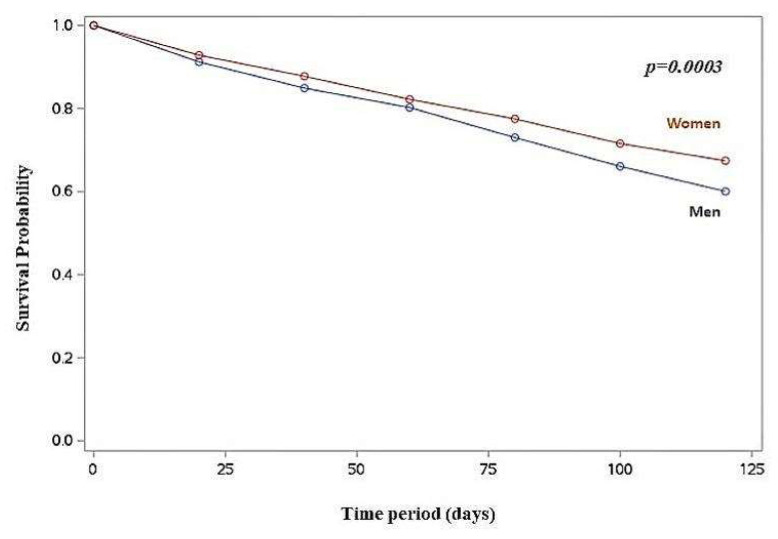
Life-table survival curves for women and men.

Kaplan–Meier survival analysis revealed a higher cumulative death incidence in men compared to women (log-rank *p* < 0.001) (Figure 1), as well as in men with RHR > 60 bpm (log-rank *p* < 0.001) and RHR > 70 bpm (log-rank *p* = 0.002), compared to women.

**Figure 2:**
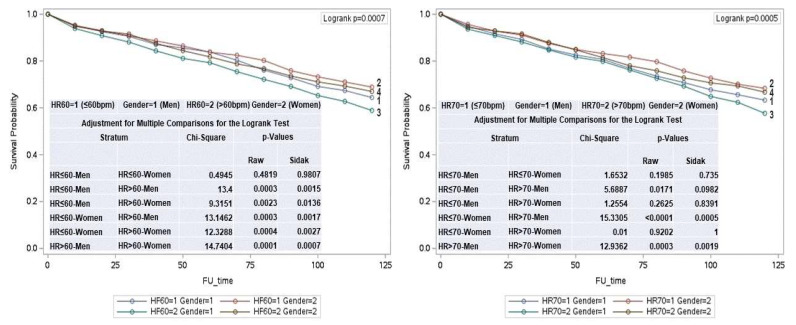
Life-table survival curves comparing women and men categorized by heart rate (HR) groups: ≤60 bpm and >60, and ≤70 bpm and >70.

In men, an RHR > 60 bpm was associated with increased mortality compared to those with an RHR of ≤60 bpm (*p* = 0.001). Conversely, in women, mortality was higher in those with an RHR of ≤ 60 bpm compared to those with an RHR of >60 bpm (*p* = 0.003). (Figure 2)

Cox proportional hazards regression analysis, adjusted for covariates with *p* < 0.25, identified age (hazard ratio [HR] = 1.01; 95% confidence interval [CI]: 1.008–1.017; *p* < 0.001), men (HR = 0.80; 95% CI: 0.71–0.90; *p* < 0.001), LVEF (HR = 0.986; 95% CI: 0.98–0.99; *p* = 0.001), and RHR (HR = 1.005; 95% CI: 1.00–1.01; *p* = 0.025) as independent death predictors. Further stratified Cox proportional hazards regression analysis indicated that age (HR = 1.01; 95% CI: 1.01–1.02; *p* = 0.002), LVEF (HR = 0.98; 95% CI: 0.97–0.99; *p* = 0.001), and RHR (HR = 1.008; 95% CI: 1.00–1.01; *p* = 0.008) were significant independent death predictors in men. For women, age emerged as the only significant predictor (HR = 1.02; 95% CI: 1.01–1.02; *p* < 0.001).

## 4. Discussion

This retrospective study investigated the association between RHR and all-cause mortality in a cohort of 2984 patients. The results revealed sex differences in mortality rates and their predictors. Men with an elevated RHR (>60 bpm) exhibited higher mortality rates compared to women. Interestingly, women with an elevated RHR (>60 or >70 bpm) had lower mortality rates than men, highlighting potential differences in the prognostic significance of RHR between sexes.

Our cohort’s baseline clinical and echocardiographic characteristics, including age, BMI, history of MI, diabetes, CKD, stroke, carvedilol dosage, hospitalization rates, and baseline LVEF, were comparable between women and men. This comparability suggests that any observed differences in outcomes are less likely to be due to baseline disparities, thereby enhancing the reliability of our findings. While both women and men started with similar baseline characteristics, the progression and outcomes followed distinct sex-influenced patterns. Women exhibited unique echocardiographic characteristics compared to men, including higher final LVEF and lower final LVDD in most HR subgroups, except for final LVEF in patients with RHR ≤ 60 bpm and >70 bpm. These differences could significantly affect disease progression, treatment response, and outcomes. The higher LVEF and lower LVDD observed in women may reflect sex-specific variations in cardiac remodeling, contractile function, and prognosis, as supported by evidence of gender differences in cardiovascular disease manifestations and outcomes [12]. Additionally, the burden of systolic and diastolic dysfunction varies by sex, with women often presenting with preserved ejection fraction [13].

The EPIC-Norfolk study demonstrated distinct patterns in HF incidence related to the RHR in apparently healthy subjects. In the overall population, HF incidence was higher for individuals with an RHR exceeding 80 bpm. Among men, a higher HF incidence was observed beginning earlier at an RHR of 71 bpm; whereas, for women, the HF incidence was more significant in the RHR of 81 to 90 bpm range when adjusted only for age. However, after applying a multivariate adjustment, the HF incidence was higher for an RHR between 81 and 90 bpm, with similar rates observed in both women and men [1]. Other studies also showed the relationship between a higher RHR and HF incidence. However, specific studies of the RHR influence in women and men with HFrEF are scarce.

Our study found that age, LVEF, and RHR were independent variables for death in men with HFrEF in sinus rhythm. In contrast, age was the only independent variable for women. The finding that an RHR was a significant predictor of mortality in men but not in women suggests that interventions aimed at reducing the RHR may need to be more aggressively pursued in men. On the other hand, the fact that age was the only significant predictor for women highlights the importance of focusing on age-related comorbidities and, possibly, different therapeutic RHR targets in female patients. In contrast, age was the only independent variable for women that was not statistically significant in the univariate analysis. However, it became an independent variable in the multivariate analysis as the joint evaluation of variables better isolates its actual effect while accounting for confounding and collinearity. We know that aging is one of the most significant factors associated with a higher incidence of heart failure. Despite population aging, the Global Burden of Disease Study 2017 reported a substantial reduction in the primary diseases causing heart failure, such as ischemic heart disease, cardiomyopathies, and myocarditis, except for hypertensive heart disease, which increased from 2007 to 2017 [14]. A UK population-based study in models standardized for age and sex also showed a decrease in the incidence of heart failure from 2002 to 2014 [15]. This overall decline was consistent across most age groups, except those ≥85 years. However, a population-based study conducted in the United States revealed a reversal in HF mortality trends, showing an increased death rate among both women and men after 2012 [16]. A key limitation of the study is its reliance on death certificate data, which may incorrectly attribute some deaths, particularly in cases where HF symptoms are similar to those of other conditions.

The sex differences in mortality observed in our study suggest that men with a higher RHR are at greater risk, whereas women appear to benefit from an RHR closer to 70 bpm. Current guidelines do not clearly define an RHR target for patients with HFrEF in sinus rhythm; however, a previous meta-analysis of 14,313 patients with HFrEF in sinus rhythm found that an RHR closer to 60 bpm was associated with lower all-cause mortality [17]. The authors of the meta-analysis suggested that achieving an RHR below 60 bpm may benefit all patients because it serves as a physiological marker of adequate beta-receptor blockade. However, the analysis did not include sex-specific findings. This gap in knowledge regarding sex-specific RHR targets provided the rationale for our study, which evaluates the impact of two RHR cutoff values—60 bpm, as suggested by the meta-analysis, and 70 bpm—on all-cause mortality. Although the mechanisms involved are unknown, several possible mechanisms could explain why an RHR was an independent variable for HFrEF in men but not women. This could be due to biological differences, such as hormonal influences on cardiovascular function and sex-specific responses to treatment and lifestyle factors [18,19,20,21]. Women and men have different autonomic nervous system responses, with men typically exhibiting higher sympathetic and lower parasympathetic activity than women. Likewise, a recent study showed different pathophysiological pathways in women and men with HF, with more significant activity of neuro-inflammatory markers in men [22]. This higher neurohumoral activity could be associated with more significant myocardial dysfunction, sympathetic activity, and RHR, and, consequently, worse prognosis in men with HFrEF. However, studies revealed conflicting results, showing that women tend to have a more significant humoral immune reaction. This heightened immune response in women is modulated by the attenuating effects of endogenous estrogen [23,24] Women generally exhibit higher levels of pro-inflammatory cytokines, such as tumor necrosis factor-alpha (TNF-α), interleukins (IL-6, IL-1β), and signaling proteins that mediate and regulate immunity and inflammation. They also show increased activation of inflammatory T cells, including Th1 and Th17 cells, which are crucial in driving inflammatory responses. Pro-inflammatory gene expression is upregulated in the female myocardium, suggesting a greater propensity for inflammatory reactions at the genetic level in heart tissue. These differences could adversely influence RHR regulation and its impact on HF development and outcome. Conversely, women might benefit from the cardioprotective effects of estrogen [25,26], which might mitigate the impact of a higher immune response and RHR on HF risk in women [27].

Our findings indicate that maintaining a resting heart rate (RHR) closest to 60 bpm may reduce mortality risk in men but not in women as lower RHR in women was associated with a higher risk of death. This disparity is likely attributable to sex-specific differences in cardiac physiology, such as reduced cardiac output in women due to smaller left ventricular cavities, which may not be adequately compensated by their relatively higher—though still reduced—left ventricular ejection fraction (LVEF), compared to men. Consequently, clinicians should consider adopting sex-specific RHR targets in the management of heart failure with reduced ejection fraction (HFrEF), potentially aiming for a slightly higher RHR (closer to 70 bpm) in women. However, further research is needed to define the optimal RHR for women with HFrEF. Strategies to address age-related risk factors should also be prioritized in female patients. Additionally, the lower mortality rates in women suggest that they benefit from different or additional protective mechanisms that could be further explored to enhance treatment approaches for men.

Cardiac implantable electronic devices are crucial in managing heart rate and rhythm in patients with HFrEF in sinus rhythm. Pacemakers help prevent bradycardia-related instability, while ICDs mitigate the risk of sudden cardiac death by detecting and treating life-threatening arrhythmias.

In our study, only a small number of patients underwent ICD implantation, which may have influenced mortality rates in both men and women. Current guidelines recommend ICD implantation for primary prevention in patients with symptomatic heart failure (NYHA class II–III) of ischemic origin, provided they have an LVEF ≤ 35% despite more than three months of optimized medical therapy, are expected to survive for more than one year with good functional status, and have not experienced a myocardial infarction within the past 40 days. For secondary prevention, ICD implantation is advised for patients who have survived a hemodynamically unstable ventricular arrhythmia, provided there are no reversible causes, and the arrhythmia did not occur within 48 h of a myocardial infarction. By appropriately selecting patients for ICD therapy, the risk of sudden death and all-cause mortality can be significantly reduced, improving long-term outcomes in HFrEF management [28,29].

### Limitations of the Study

This study has several limitations. Our study’s observational design identified associations between variables. However, it cannot establish causation due to the potential influence of confounding factors and biases, which may affect the findings and preclude definitive causal conclusions. Furthermore, the specific cohort characteristics may limit the generalizability of the results. It is important to note that unmeasured confounding factors could also significantly impact the interpretation of the findings, highlighting the need for further research in this area. It is a retrospective study in a specialized tertiary care center where selection biases may occur, including patients with a more complex clinical picture. Prognostic risk factors for HFrEF, such as socioeconomic status, exercise frequency and intensity, education level, and psychological well-being, were unavailable. An adequate definition of symptoms is missing, especially dyspnea and NYHA functional class, as well as other variables associated with a worse prognosis, such as ventricular arrhythmia and a 6-min walk test. We were also unable to detail the cause of death adequately. Our analysis included cardiac and non-cardiac causes, including the deaths from COVID-19, which occurred between the pandemic months of March and September 2020. However, the impact of the COVID-19 epidemic on all-cause mortality in our study population remains uncertain. Nevertheless, a previous study conducted in our general population from a highly populated city found that the epidemic did not have the expected increased effect on patients with cardiovascular disease [30]. Finally, adequate information regarding drug treatment and dosages needs to be included. However, our center advocates that HF treatment be as close as possible to current guidelines, as shown by the high percentage of HF common medications in the tables. The significant improvement in LVEF observed in our cohort also emphasizes the potential benefits of optimized medical therapy and lifestyle modifications in enhancing cardiac function over time. Additionally, the study did not explore the potential impact of hormonal status, menopausal status, or hormone replacement therapy on HF outcomes, which could be relevant factors in sex-based differences.

## 5. Conclusions

In conclusion, this study highlights the critical role of the RHR as a predictor of mortality in patients with HFrEF, emphasizing distinct sex-specific differences. An elevated RHR appears to confer a greater risk in men, underscoring the importance of targeted interventions to maintain an RHR near 60 bpm. In contrast, our findings suggest that women may derive optimal benefit from a slightly higher RHR, closer to 70 bpm, supporting the need for individualized heart rate management strategies tailored to sex-specific physiological responses. Optimal heart rate control in HFrEF requires a personalized, evidence-based approach that integrates pharmacological therapy, lifestyle modifications, and, when appropriate, device-based interventions. Given the well-established impact of the heart rate on cardiovascular outcomes, maintaining sex-specific RHR targets—approximately 60 bpm for men and 70 bpm for women—should be a key consideration in clinical management. Implementing individualized strategies that account for sex-related differences in heart rate regulation may contribute to improved prognosis and overall treatment efficacy in patients with HFrEF. This could be accomplished by pharmacological interventions, particularly beta-blockers such as carvedilol, bisoprolol, and metoprolol succinate, which remain the cornerstone of heart rate modulation in HFrEF. These agents exert their therapeutic effects by antagonizing beta-adrenergic receptors, attenuating sympathetic drive, reducing myocardial oxygen demand, and improving ventricular efficiency. More recently, ivabradine is an option for patients whose target RHR was not achieved with beta-blockers. Additionally, non-pharmacological strategies play a complementary role in heart rate management. Regular physical activity, weight optimization, and dietary modifications contribute to cardiovascular health and may support heart rate control.

Future research should focus on interventional strategies to optimize RHR and improve outcomes, especially in men who appear to be at a higher risk. Understanding the underlying mechanisms of these sex differences could lead to more personalized and effective treatments for both men and women.

## Figures and Tables

**Table 1:** Clinical characteristics, medications used, surgical interventions, and echocardiographic data for all patients, women and men with heart failure with reduced ejection fraction in sinus rhythm.

	All Patients	Men	Women	*p*
	N = 2984	N = 1922 (64.4)	N = 1062 (35.6)	
Age (Years)	61 ± 13.8	60.8 ± 13.6	61.3 ± 14.1	0.271
Body mass index (kg/m^2^)	28.8 ± 23.1	29.3 ± 26.5	28.0 ± 15.0	0.071
Resting heart rate (bpm)	72.0 ± 11.6	72.1 ± 11.8	71.8 ± 11.3	0.475
Myocardial infarction (%)	456 (15.3)	302 (15.7)	154 (14.5)	0.378
Diabetes (%)	520 (17.4)	334 (17.4)	186 (17.5)	0.925
Chronic kidney disease (%)	359 (12)	239 (12.4)	120 (11.3)	0.361
Stroke (%)	146 (4.9)	88 (4.6)	58 (5.5)	0.284
ACEI or ARB (%)	2347 (78.7)	1497 (77.9)	850 (80.0)	0.170
Beta-blocker (%)	2759 (92.5)	1773 (92.3)	986 (92.8)	0.587
Carvedilol (mg/day)	40.7 ± 16.2	40.6 ± 16.2	40.9 ± 16.1	0.752
Spironolactone (%)	1847 (61.9)	1143 (59.5)	704 (66.3)	<0.001
Furosemide (%)	1920 (64.4)	1206 (62.8)	714 (67.2)	0.015
Hydrochlorothiazide (%)	422 (14.2)	267 (13.9)	155 (14.6)	0.601
CABG (%)	184 (6.2)	131 (6.8)	53 (5.0)	0.047
PCI (%)	95 (3.2)	78 (4.1)	17 (1.6)	<0.001
Pacemaker (%)	220 (7.4)	133 (6.9)	87 (8.2)	0.206
ICD (%)	157 (5.3)	120 (6.2)	37 (3.5)	0.001
CRT (%)	233 (7.8)	141 (7.3)	92 (8.7)	0.193
Transplant (%)	139 (4.7)	84 (4.4)	55 (5.2)	0.317
Hospitalization (%)	1701 (57.0)	1095 (57.0)	606 (57.1)	0.962
LVEF basal (%)	29.6 ± 6.6	29.5 ± 6.7	29.9 ± 6.4	0.106
LVEF final (%)	37.1 ± 13.1	36.7 ± 12.9	38.0 ± 13.4	0.016
LVDD basal (mm)	64.7 ± 8.5	65.4 ± 8.7	63.3 ± 7.9	<0.001
LVDD final (mm)	62.4 ± 10.4	63.1 ± 10.6	61.2 ± 9.9	<0.001
Death (%)	1229 (41.2)	839 (43.6)	390 (36.7)	<0.001

Values are means ± standard deviations, and in parentheses, mean percentages. ACEI: angiotensin-converting enzyme inhibitor; ARB: angiotensin receptor blocker; CABG: coronary artery bypass graft; CRT: cardiac resynchronization therapy; LVDD: left ventricular diastolic diameter; LVEF: left ventricular ejection fraction; ICD: implantable cardioverter-defibrillator; PCI: percutaneous coronary intervention.

**Table 2:** Clinical characteristics, medications used, surgical interventions, and echocardiographic data for all patients and patients with ≤60> bpm and ≤70> bpm with heart failure with reduced ejection fraction in sinus rhythm.

	All Patients	HR ≤ 60	HR > 60	HR ≤ 70	HR > 70
	N = 2984	N = 122 (4.1)	N = 2862 (95.9)	N = 1267 (42.5)	N = 1717 (57.5)
Age (Years)	61 ± 13.8	60.5 ± 13.3	61 ± 13.8	61.3 ± 14.1	60.8 ± 13.6
Women (%)	1062 (35.6)	38 (31.2)	1024 (35.8)	458 (36.2)	604 (35.2)
Body mass index (kg/m^2^)	28.8 ± 23.1	28.8 ± 29.6	28.8 ± 22.8	28.5 ± 26.7	29.1 ± 20
Resting heart rate (bpm)	72.0 ± 11.6	55.7 ± 2.1	72.7 ± 11.4 *	62.2 ± 3.7	79.2 ± 10.1 *
Myocardial infarction (%)	456 (15.3)	19 (15.6)	437 (15.3)	205 (16.2)	251 (14.6)
Diabetes (%)	520 (17.4)	18 (14.8)	502 (17.5)	224 (17.7)	296 (17.2)
Chronic kidney disease (%)	359 (12)	17 (13.9)	342 (12)	158 (12.5)	201 (11.7)
Stroke (%)	146 (4.9)	4 (3.3)	142 (5)	65 (5.1)	81 (4.7)
ACEI or ARB (%)	2347 ()	90 (73.8)	2257 (78.9)	1001 (79.0)	1346 (78.4)
Beta-blocker (%)	2759 (92.5)	107 (87.7)	2652 (92.7) *	116 (92.1)	1593 (92.8)
Carvedilol (mg/day)	40.7 ± 16.2	42.4 ± 15.2	40.7 ± 16.2	40.8 ± 16.2	40.7 ± 16.2
Spironolactone (%)	1847 (61.9)	67 (54.9)	1780 (62.2)	777 (61.4)	1070 (62.3)
Furosemide (%)	1920 (64.4)	71 (58.2)	1849 (64.6)	800 (63.2)	1120 (65.2)
Hydrochlorothiazide (%)	422 (14.2)	19 (15.6)	403 (14.1)	181 (14.3)	241 (14.0)
CABG (%)	184 (6.2)	10 (8.2)	174 (6.1)	86 (6.8)	98 (5.7)
PCI (%)	95 (3.2)	1 (1.0)	94 (3.3)	45 (3.6)	50 (2.9)
Pacemaker (%)	220 (7.4)	10 (8.2)	210 (7.3)	102 (8.1)	118 (6.9)
ICD (%)	157 (5.3)	9 (7.4)	148 (5.2)	78 (6.2)	79 (4.6)
CRT (%)	233 (7.8)	8 (6.6)	225 (7.9)	89 (7.0)	144 (8.4)
Transplant (%)	139 (4.7)	11 (9.0)	128 (4.5)	59 (4.7)	80 (4.7)
Hospitalization (%)	1701 (57.0)	71 (58.2)	1630 (57.0)	430 (33.9)	601 (35)
LVEF basal (%)	29.6 ± 6.6	29.5 ± 6.8	29.6 ± 6.6	29.7 ± 6.6	29.6 ± 6.6
LVEF final (%)	37.1 ± 13.1	37.9 ^±^ 14.6	37.1 ± 13	37.4 ± 13.2	36.9 ± 13
LVDD basal (mm)	64.7 ± 8.5	66.2 ± 8.7	64.6 ± 8.5 *	64.5 ± 8.6	64.8 ± 8.4
LVDD final (mm)	62.4 ± 10.4	62.6 ± 10.5	62.4 ± 10.4	61.9 ± 10.4	62.8 ± 10.4
Death (%)	1229 (41.2)	48 (39.3)	1181 (41.3)	495 (39.1)	734 (42.8) *

* *p* < 0.05; Values mean means ± standard deviations, and mean percentages in parentheses. ACEI: angiotensin-converting enzyme inhibitor; ARB: angiotensin receptor blocker; CABG: coronary artery bypass graft; CRT: cardiac resynchronization therapy; LVDD: left ventricular diastolic diameter; LVEF: left ventricular ejection fraction; ICD: implantable cardioverter-defibrillator; PCI: percutaneous coronary intervention.

**Table 3:** Clinical characteristics, medications used, surgical interventions, and echocardiographic data for all patients and patients with ≤60> bpm with heart failure with reduced ejection fraction in sinus rhythm.

	HR ≤ 60 N = 122(4.1)		*p*	HR > 60 N = 2862(95.9)		*p*
	Men N = 84 (68.9)	Women N = 38 (31.1)		Men N = 1838 (64.2)	Women N = 1024 (35.8)	
Age (Years)	58.4 ± 13.5	65 ± 11.9	0.011	60.9 ± 13.6	61.2 ± 14.2	0.533
Body mass index (kg/m^2^)	30.4 ± 35.5	25.1 ± 4.0	0.178	29.3 ± 26	28.1 ± 15.2	0.115
Resting heart rate (bpm)	55.6 ± 2.1	56.1 ± 2.1	0.196	72.9 ± 11.5	72.4 ± 11.1	0.267
Myocardial infarction (%)	14 (16.7)	5 (13.2)	0.621	288 (15.7)	149 (14.6)	0.425
Diabetes (%)	12 (14.3)	6 (15.8)	0.828	322 (17.5)	180 (17.6)	0.968
Chronic kidney disease (%)	11 (13.1)	6 (15.8)	0.691	228 (12.4)	114 (11.1)	0.315
Stroke (%)	2 (2.4)	2 (5.3)	0.408	86 (4.7)	56 (5.5)	0.351
ACEI or ARB (%)	66 (78.6)	24 (63.2)	0.073	1431 (77.9)	826 (80.7)	0.078
Beta-blocker (%)	74 (88.1)	33 (86.8)	0.845	1699 (92.5)	953 (93.1)	0.569
Carvedilol (mg/day)	43.4 ± 14.4	39.8 ± 17.1	0.341	40.5 ± 16.3	40.9 ± 16.1	0.599
Spironolactone (%)	48 (57.1)	19 (50.0)	0.463	1095 (59.6)	685 (66.9)	<0.001
Furosemide (%)	51 (60.7)	20 (52.6)	0.402	1155 (62.9)	694 (67.8)	0.009
Hydrochlorothiazide (%)	14 (16.7)	5 (13.2)	0.621	253 (13.8)	150 (14.7)	0.519
CABG (%)	8 (9.5)	2 (5.3)	0.427	123 (6.7)	51 (5.0)	0.066
PCI (%)	1 (1.2)	0 (0)	0.499	77 (4.2)	17 (1.7)	<0.001
Pacemaker (%)	7 (8.3)	3 (7.9)	0.935	126 (6.9)	84 (8.2)	0.188
ICD (%)	8 (9.5)	1 (2.6)	0.177	112 (6.1)	36 (3.5)	0.003
CRT (%)	5 (6.0)	3 (7.9)	0.271	136 (7.4)	89 (8.7)	0.216
Transplant (%)	5 (6.0)	6 (15.8)	0.079	79 (4.3)	49 (4.8)	0.548
Hospitalization (%)	52 (61.9)	19 (50.0)	0.217	1043 (56.8)	587 (57.3)	0.765
LVEF basal (%)	29.3 ± 7.1	30.1 ± 6.4	0.523	29.5 ± 6.7	29.9 ± 6.4	0.130
LVEF final (%)	39.2 ± 15	34.8 ± 13.3	0.162	36.5 ± 12.8	38.1 ± 13.4	0.005
LVDD basal (mm)	67.1 ± 9.4	64.2 ± 6.7	0.055	65.4 ± 8.7	63.2 ± 7.9	<0.001
LVDD final (mm)	63.4 ± 10.8	60.7 ± 9.8	0.320	63.1 ± 10.6	61.2 ± 9.9	<0.001
Death (%)	32 (38.1)	16 (42.1)	0.675	807 (43.9)	374 (36.5)	<0.001

Values are means ± standard deviations, and in parentheses, mean percentages. ACEI: angiotensin-converting enzyme inhibitor; ARB: angiotensin receptor blocker; CABG: coronary artery bypass graft; CRT: cardiac resynchronization therapy; LVDD: left ventricular diastolic diameter; LVEF: left ventricular ejection fraction; ICD: implantable cardioverter-defibrillator; PCI: percutaneous coronary intervention.

**Table 4:** Clinical characteristics, medications used, surgical interventions, and echocardiographic data for all patients and patients with ≤70> bpm with heart failure with reduced ejection fraction in sinus rhythm.

	HR ≤ 70 N = 1267 (42.5)			HR > 70 N = 1717 (57.5)		
	Men N = 809 (62.9)	Women N = 458 (36.1)	*p*	Men N = 1113 (64.8)	Women N = 604 (35.2)	*p*
Age (Years)	61 ± 13.8	61.8 ± 14.6	0.319	60.6 ± 13.5	61 ± 13.8	0.569
BMI (kg/m^2^)	29 ± 30.3	27.6 ± 18.8	0.308	29.5 ± 23.4	28.2 ± 11.2	0.111
Resting heart rate (bpm)	62.2 ± 3.8	62.3 ± 3.5	0.692	79.3 ± 10.3	79.0 ± 9.7	0.543
Myocardial infarction (%)	141 (17.4)	64 (14)	0.109	161 (14.5)	90 (14.9)	0.807
Diabetes (%)	146 (18.1)	78 (17)	0.649	188 (16.9)	108 (17.9)	0.604
Chronic kidney disease (%)	101 (12.5)	57 (12.5)	0.984	138 (12.4)	63 (10.4)	0.226
Stroke (%)	38 (4.7)	27 (5.9)	0.353	50 (4.5)	31 (5.1)	0.550
ACEI or ARB (%)	640 (79.1)	361 (78.8)	0.903	857 (77.0)	489 (81.0)	0.057
Beta-blocker (%)	742 (91.8)	424 (92.6)	0.637	1031 (92.6)	562 (93.1)	0.752
Carvedilol (mg/day)	40.6 ± 16.3	41.1 ± 16.0	0.631	40.7 ± 16.2	40.7 ± 16.2	0.998
Spironolactone (%)	484 (59.9)	293 (64.0)	0.153	659 (59.2)	411 (68.1)	<0.001
Furosemide (%)	497 (61.5)	303 (66.2)	0.099	709 (63.7)	411 (68.1)	0.071
Hydrochlorothiazide (%)	109 (13.5)	72 (15.7)	0.276	158 (14.2)	83 (13.7)	0.796
CABG (%)	64 (7.9)	22 (4.8)	0.035	67 (6.0)	31 (5.1)	0.447
PCI (%)	37 (4.6)	8 (1.8)	0.009	41 (3.7)	9 (1.5)	0.010
Pacemaker (%)	52 (6.4)	50 (10.9)	0.005	81 (7.3)	37 (6.1)	0.365
ICD (%)	63 (7.8)	15 (3.3)	0.001	57 (5.1)	22 (3.7)	0.165
CRT (%)	59 (7.3)	30 (6.6)	0.619	82 (7.4)	62 (10.3)	0.038
Transplant (%)	35 (4.3)	24 (5.2)	0.458	49 (4.4)	31 (5.1)	0.496
Hospitalization (%)	468 (57.8)	255 (55.7)	0.453	627 (56.3)	351 (58.1)	0.477
LVEF basal (%)	29.4 ± 6.7	30.1 ± 6.3	0.060	29.5 ± 6.7	29.7 ± 6.5	0.607
LVEF final (%)	36.7 ± 13.1	38.6 ± 13.4	0.024	36.6 ± 12.8	37.5 ± 13.4	0.237
LVDD basal (mm)	65.4 ± 8.7	63.0 ± 8.1	<0.001	65.5 ± 8.7	63.5 ± 7.8	<0.001
LVDD final (mm)	62.7 ± 10.5	60.6 ± 10.2	0.007	63.4 ± 10.7	61.7 ± 9.7	0.008
Death (%)	330 (40.8)	165 (36)	0.095	509 (45.7) *	225 (37.3)	0.001

Values are means ± standard deviations, and in parentheses, means percentages. ACEI: angiotensin-converting enzyme inhibitor; ARB: angiotensin receptor blocker; CABG: coronary artery bypass graft; CRT: cardiac resynchronization therapy; LVDD: left ventricular diastolic diameter; LVEF: left ventricular ejection fraction; ICD: implantable cardioverter-defibrillator; PCI: percutaneous coronary intervention. * *p* < 0.05 for the comparison of death percentage between men with RHR ≤ 70 bpm and men with RHR > 70 bpm.

## Data Availability

Data are unavailable due to privacy reasons.

## Abbreviations

The following abbreviations are used in this manuscript:

RHR	Resting heart rate
HFrEF	Heart failure with reduced ejection fraction
HF	Heart failure
LVEF	Left ventricular ejection fraction
LVDD	Left ventricular diastolic diameters
BMI	Body mass index
CKD	Chronic kidney disease
ACEI	Angiotensin-converting enzyme inhibitor
ARB	Angiotensin receptor blocker
CABG	Coronary artery bypass graft
CRT	Cardiac resynchronization therapy
ICD	Implantable cardioverter-defibrillator
PCI	Percutaneous coronary intervention
